# Towards Radiolabeled EGFR-Specific Peptides: Alternatives to GE11

**DOI:** 10.3390/ph16020273

**Published:** 2023-02-11

**Authors:** Benedikt Judmann, Björn Wängler, Ralf Schirrmacher, Gert Fricker, Carmen Wängler

**Affiliations:** 1 Biomedical Chemistry, Clinic of Radiology and Nuclear Medicine, Medical Faculty Mannheim, Heidelberg University, 68167 Mannheim, Germany; 2 Molecular Imaging and Radiochemistry, Clinic of Radiology and Nuclear Medicine, Medical Faculty Mannheim, Heidelberg University, 68167 Mannheim, Germany; 3 Department of Oncology, Division of Oncological Imaging, University of Alberta, Edmonton, AB T6G 1Z2, Canada; 4 Institute of Pharmacy and Molecular Biotechnology, University of Heidelberg, 69120 Heidelberg, Germany

**Keywords:** EGFR, peptidic radiotracers, ^68^Ga, GE11, D4, P1, P2, CPP, QRH, EGBP, Pep11 peptides, truncated hEGF

## Abstract

The human epidermal growth factor receptor (EGFR) is closely related to several cancer-promoting processes and overexpressed on a variety of tumor types, rendering it an important target structure for the imaging and therapy of several malignancies. To date, approaches to develop peptidic radioligands able to specifically address and visualize EGFR-positive tumors have been of limited success. Most of the attempts were based on the lead GE11, as this peptide was previously described to be a highly potent EGFR-specific agent. However, since it has recently been shown that GE11 exhibits an insufficient affinity to the EGFR in monomeric form to be suitable as a basis for the development of tracers based on it, in the present work we investigated which other peptides might be suitable as lead structures for the development of EGFR-specific peptidic radiotracers. For this purpose, we developed ^68^Ga-labeled radioligands based on the peptides D4, P1, P2, CPP, QRH, EGBP and Pep11, having been described before as EGFR-specific. In addition, we also tested three truncated versions of the endogenous EGFR ligand hEGF (human epidermal growth factor) with respect to their ability to specifically target the EGFR with high affinity. Therefore, chelator-modified labeling precursors of the mentioned peptides were synthesized, radiolabeled with ^68^Ga and the obtained radioligands were evaluated for their hydrophilicity/lipophilicity, stability against degradation by human serum peptidases, in vitro tumor cell uptake, and receptor affinity in competitive displacement experiments on EGFR-positive A431 cells. Although all NODA-GA-modified (NODA-GA: (1,4,7-triazacyclononane-4,7-diyl)diacetic acid-1-glutaric acid) labeling precursors could be obtained more or less efficient in yields between 5 and 74%, the ^68^Ga-radiolabeling proved to be unsuccessful for two of the three truncated versions of hEGF ([^68^Ga]Ga-**8** and [^68^Ga]Ga-**9**), producing several side-products. For the other agents [^68^Ga]Ga-**1**–[^68^Ga]Ga-**7**, [^68^Ga]Ga-**10** and [^68^Ga]Ga-**11**, high radiochemical yields and purities of ≥98% and molar activities of up to 114 GBq/µmol were obtained. In the assay investigating the radiopeptide susceptibilities against serum peptidase degradation, the EGBP-based agent demonstrated a limited stability with a half-life of only 66.4 ± 3.0 min, whereas the other tracers showed considerably higher stabilities of up to an 8000 min half-life. Finally, all radiotracer candidates were evaluated in terms of tumor cell internalization and receptor binding potential on EGFR-positive A431 cell. In these experiments, all developed agents failed to show an EGFR-specific tumor cell uptake or a relevant EGFR-affinity. By contrast, the positive controls tested under identical conditions, [^125^I]I-hEGF and hEGF demonstrated the expected high EGFR-specific tumor cell uptake (33.6% after 1 h, being reduced to 1.9% under blocking conditions) and affinity (IC_50_ value of 15.2 ± 3.3 nM). Thus, these results indicate that none of the previously described peptidic agents developed for EGFR targeting appears to be a reasonable choice as a lead structure for the development of radiopeptides for targeting of EGFR-positive tumors. Likewise, the tested truncated variants of the endogenous hEGF do not seem to be promising alternatives for this purpose.

## 1. Introduction

For positron emission tomography (PET), which is a highly sensitive molecular imaging technique, biomolecules that feature a highly specific accumulation at the target site with high affinity are particularly suitable as targeting vectors and carriers for the radionuclide. In oncological imaging, these are mainly receptor-affine compounds which bind to receptors that are overexpressed on the tumor cell surface.

A receptor of high relevance for the imaging of malignant transformations is the EGFR (also termed as HER1) which belongs to the EGF receptor family, all of which have important regulatory functions in different physiological processes such as cell differentiation, cell cycle progression, proliferation, apoptosis and protein transcription [1,2,3]. The EGFR is, however, also closely related to several cancer-promoting processes, namely tumor cell proliferation, angiogenesis, increased cell motility, metastatic growth and apoptosis-suppression [3,4,5]. As such, it is overexpressed on a variety of tumor types [1,6,7,8], underlining its importance as target structure for tumor imaging and therapy.

For EGFR-targeted delivery of radionuclides to tumors, antibodies and fragments thereof are well-studied [5]. Although they exhibit excellent target specificity and affinity, antibodies also exhibit several drawbacks when being applied as basis of radiopharmaceuticals, such as slow in vivo pharmacokinetics combined with slow background clearance as well as limited tissue penetration. In contrast, peptides, with their much faster pharmacokinetics, small size that does not limit tissue penetration, high target affinity and binding specificity, easy synthetic availability, and good ability to be chemically modified in a defined way enabling tailoring of the molecule, possess several advantages compared to protein-based agents. Thus, peptides are commonly used as tumor targeting vectors for oncological imaging by PET.

**Figure 1:**
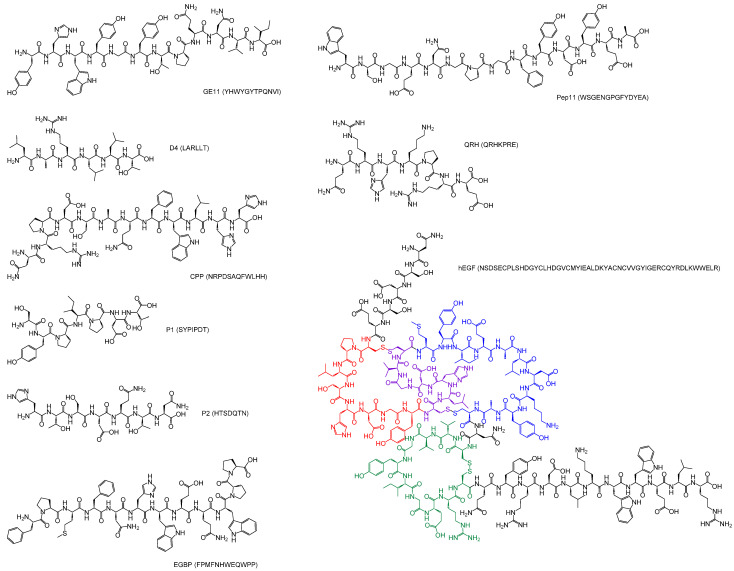
Structures of the peptide leads GE11, D4, CPP, P1, P2, EGBP, Pep11, QRH and hEGF of which chelator-modified and radiolabeled analogs were developed of and tested for their potential to be applied as agents for EGFR-specific tumor targeting. In the structure of hEGF, the three loops of the peptide are colored in red (A loop), blue (B loop) and green (C loop), while the part of the peptide belonging to both the A and the B loop is depicted in purple.

As a result, several attempts have been made in recent years to develop a positron emitter-labeled EGFR-specific peptide for use in PET imaging of EGFR-overexpressing tumors. In this regard, most approaches were based on the peptide GE11 (Figure 1) as a tumor targeting vector [9,10,11,12,13,14], which has been described to exhibit a very good EGFR targeting ability [4,15]. However, none of these approaches to use GE11 as basis for EGFR-specific PET tracers have resulted in a radiopharmaceutical with high potential yet. This can be attributed to the fact that the GE11 peptide itself possesses an insufficient affinity to the EGFR being reflected in an only limited, if not absent, EGFR-specific tumor uptake and an only suboptimal tumor visualization ability of the corresponding imaging agents developed [11,12,14]. 

Therefore, with the present work, we aimed to identify a peptidic alternative to GE11 being an EGFR-specific agent and, thus, be a suitable basis for the future development of a radiolabeled agent applicable in PET imaging of EGFR-positive tumors as the long-term goal. For this purpose, we screened the literature for peptidic structures for which an EGFR-specific interaction has been described. The most important of these is D4 (Figure 1) being identified using a rational in silico receptor crystal structure-based design approach [16] and for which, as for GE11, a number of studies are available demonstrating an interaction of the peptide with the EGFR [16,17,18]. Due to this, D4 has been used as basis for the development of ^99m^Tc-labeled EGFR-specific SPECT imaging agents (SPECT: single photon emission computed tomography), although with similar results as GE11-based tracers [19,20,21]. 

Apart from GE11 and D4, several other peptides, namely CPP, P1, P2, EGBP, Pep11 and QRH (Figure 1) have been identified, mostly using phage display, and described as EGFR-specific binders over the last years [22,23,24,25,26,27,28]. Thus, these peptides, together with GE11 and D4, have also been investigated here with regard to their suitability as EGFR-specific targeting vectors.

In addition, three truncated, cyclic peptide fragments of hEGF (human epidermal growth factor (Figure 1), endogenous EGFR ligand, binding to the receptor with high affinity [29,30]) were studied under the same conditions in order to investigate if one of these offers a high potential for EGFR targeting. As known from the crystal structure of the complex of human EGFR and hEGF, all three loops of the peptide interact with different sites/domains of the EGFR [29]. Furthermore, a truncated version of mouse EGF, based on the EGF_20–31_ sequence, was shown before to exhibit a low but detectable affinity to the respective human EGFR [31]. Thus, truncated versions of the human peptide, consisting of these different loops, could, in principle, be valuable targeting vectors still enabling tight EGFR binding while exhibiting a higher stability due to decreased size and thus susceptibility to degradation. Truncated peptides of endogenous ligands have often shown before to be valuable alternatives to their endogenous leads, exhibiting a comparably higher stability while being able to retain receptor specificity and, to a large extent, also receptor affinity [32].

## 2. Results and Discussion

As detailed before, chelator-modified ligands based on the peptides GE11, D4, CPP, P1, P2, EGBP, Pep11, QRH and the three loops of hEGF were to be developed here and tested for their ability to specifically interact with the EGFR with high affinity. Furthermore, their radiolabeling with ^68^Ga^3+^ should be demonstrated, and the log*_D_*_(7.4)_ of the radiolabeled agents as well as their stability against human peptidase degradation should be determined. The ability of the developed agents to interact with their target was to be determined by cell uptake studies on the one hand, and on the other hand by competitive displacement assays.

### 2.1. General Radiotracer Design and Synthesis of the Peptidic Labeling Precursors ***1***–***11***

First, the peptidic radiolabeling precursors based on the mentioned peptide leads were synthesized. As the radiolabeling should be carried out with the commonly used and clinically relevant positron emitter ^68^Ga^3+^ [33,34], a NODA-GA chelator introduced into the peptides for efficient and stable introduction of the radiometal ion [35].

Further, the peptidic structures were modified with a short PEG_5_ linker between the chelator and the receptor-affine molecules, resulting in a spatial distance between the peptidic binder and the radiometal-carrying complex, and to reduce a potential interference of the latter on peptide-receptor-binding. The position in which the PEG_5_-NODA-GA-building block was introduced into the peptide structure was chosen based on the data of the original literature where the peptides GE11, D4, P1, P2, CPP, QRH, EGBP and Pep11 as well as their chemical modification for different purposes were described. In these studies, the peptides were modified either at the *C*-terminal or the *N*-terminal end and still found to be potent candidates for specific EGFR-interaction [4,16,26,27,28,36]. In case of GE11, D4, P1 and P2, a *N*-terminal modification was applied whereas for CPP, QRH, EGBP and Pep11, a *C*-terminal derivatization was preferential. In case of the truncated derivatives of hEGF, a C-terminal modification was chosen as well.

**Figure 2:**
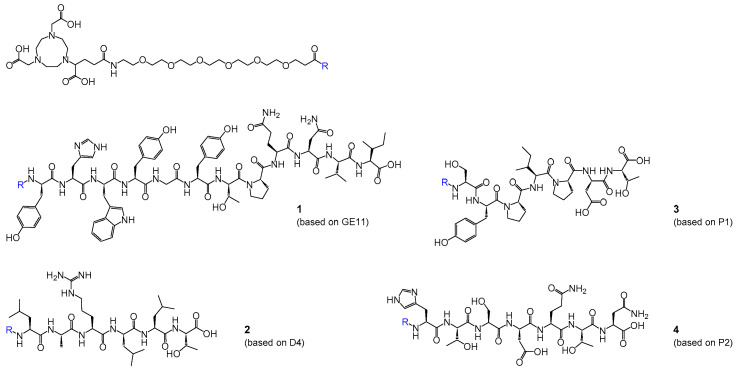
Structures of the *N*-terminally PEG_5_-NODA-GA-modified labeling precursors based on the peptides GE11 (**1**), D4 (**2**), P1 (**3**) and P2 (**4**).

**Figure 3:**
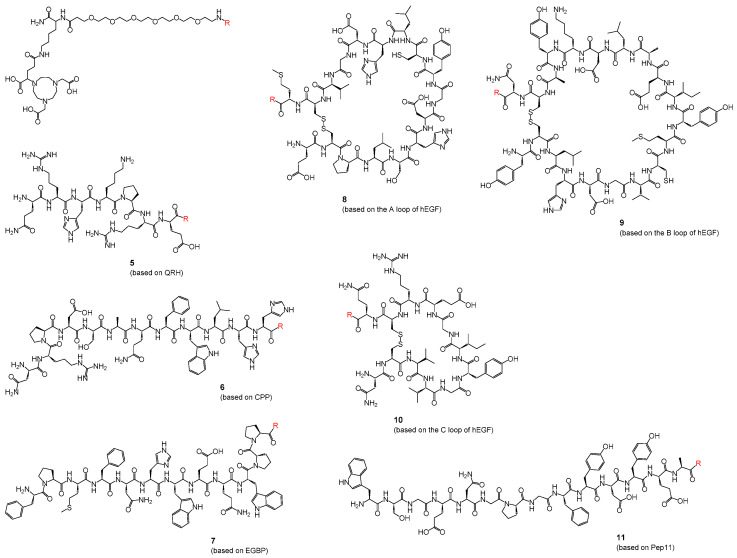
Structures of the *C*-terminally PEG_5_-NODA-GA-modified labeling precursors based on the peptides QRH (**5**), CPP (**6**), EGBP (**7**), the three loops of the hEGF (**8**–**10**) and Pep11 (**11**).

The structures of the labeling precursors based on the considerations outlined before are summarized in Figure 2 and Figure 3.

The synthesis of the labeling precursors followed standard solid phase peptide synthesis (SPPS) protocols using the Fmoc-strategy [37]. In brief, the peptide derivatives were obtained by building the linear sequences on commercially available resins by successive conjugation of the *N*_α_-Fmoc- and side chain-protected amino acid building blocks, Fmoc-NH-PEG_5_-COOH and the chelator (*R*)-NODA-GA(*^t^*Bu)_3_. For this purpose, the *N*_α_-amino functionalities were deprotected by removal of the Fmoc-protecting groups using 50% piperidine in DMF (dimethylformamide) (*v*/*v*) for 2 + 5 min. Afterwards, the next amino acid—being activated using HBTU (2-(1*H*-benzotriazol-1-yl)-1,1,3,3-tetramethyluronium hexafluorophosphate) and DIPEA (*N,N*-diisopropylethylamine) as coupling agent and base—was conjugated until the peptide sequence was complete. These conjugation reactions were performed under ultrasound-assistance using a conventional ultrasonic bath as we and others found this method to be able to substantially reduce reactants as well as conjugation time during peptide syntheses [14,38,39]. For *N*_ε_-side chain Mtt-deprotection (Mtt: 4-methyltrityl), the resins were incubated with 1% TFA (trifluoroacetic acid) in DCM before the chelating agent NODA-GA was conjugated in form of its fully protected (*R*)-NODA-GA(*^t^*Bu)_3_ derivative using PyBOP (Benzotriazole-1-yl-oxy-tris-pyrrolidino-phosphonium hexafluorophosphate) as coupling agent in order to increase coupling efficiency. Cyclizations by dithiol-formation were accomplished using *N*-chlorosuccinimide (NCS) in combination with Mmt-protected cysteines (Mmt: monomethoxytrityl) which were first side chain-deprotected by incubation of the resin with 2% TFA in DCM before the cyclization was performed. The removal of acid-labile protecting groups and simultaneous cleavage of the chelator-modified peptides from the resin was accomplished by incubating the resins with a mixture of TFA, TIS (triisopropylsilane) and H_2_O (95/2.5/2.5, *v*/*v*/*v*) for 3 h at ambient temperature and subsequent purification by semipreparative HPLC.

**Scheme 1:**
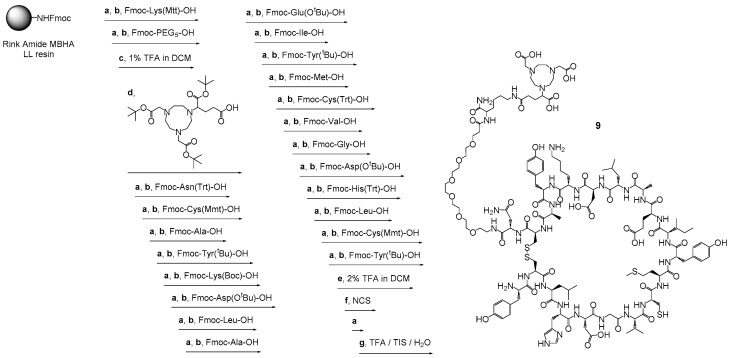
Schematic depiction of the synthesis strategy of the labeling precursors **1**–**11** exemplified for **9**. Reaction conditions: (**a**) cleavage of Fmoc-protecting group: piperidine/DMF (1/1, *v*/*v*), 2 + 5 min; (**b**) activation of amino acid: 2.0 equiv. amino acid derivative, 2.0 equiv. DIPEA, 1.9 equiv. HBTU in DMF, 2 min, 15 min conjugation; (**c**) mildly acidic cleavage of lysine *N*_ε_-Mtt protecting group: DCM/TFA (99/1, *v*/*v*), 30–45 min; (**d**) activation of NODA-GA(*^t^*Bu)_3_: 2.0 equiv., 2.0 equiv. DIPEA, 1.9 equiv. PyBOP in DMF, 2 min, 30 min conjugation; (**e**) mildly acidic cleavage of cysteine Mmt protecting group: DCM/TFA (98/2, *v*/*v*), 45 min; (**f**) cyclization by dithiol-formation using 1.05 equiv. NCS in DMF, 15 min; (**g**) cleavage of peptide from resin and simultaneous deprotection of side chain functional groups: TFA/TIS/H_2_O (95/2.5/2.5, *v*/*v*/*v*), 3 h.

The course of synthesis of the peptidic labeling precursors is exemplified for compound **9** in Scheme 1. The synthesis of the other compounds **1**–**8**, **10** and **11** was performed analogously.

In general, the peptide precursors were obtained without relevant difficulties in good isolated yields of 21–74% after purification. However, the syntheses of two of the precursors, namely **9** and **10**, were problematic, as the agents could only be isolated in comparably low yields of 5 and 10%, respectively. This was due to difficulties in the cyclization of the peptides via the respective cysteine residues, which proceeded smoothly in **8** but required optimization of the reaction conditions in **9** and **10** to afford the cyclic products. First, a conventional deprotection and cyclization strategy was applied for this purpose, being based on acetamidomethyl (Acm)-protected cysteines which can usually be efficiently and selectively deprotected on resin and simultaneously oxidized to the dithiol-bridged peptides using thallium(III)-trifluoroacetate [40]. However, no product formation was observed using this standard procedure for **9** and **10,** and the cyclization using iodine instead of the thallium salt for oxidation was not successful. In contrast, the approach to use monomethoxytrityl (Mmt)-protected cysteines, which can be deprotected under mildly acidic conditions followed by cyclization using the oxidizing agent *N*-chlorosuccinimide (NCS) [41,42], proved to be successful, although the achievable yields were not optimal.

### 2.2. ^68^Ga-Radiolabeling of ***1***–***11*** and Investigation of [^68^Ga]Ga-***1***–[^68^Ga]Ga-***11*** with Regard to log_D(7.4)_ and Stability towards Serum Peptidase Degradation

**Figure 4:**
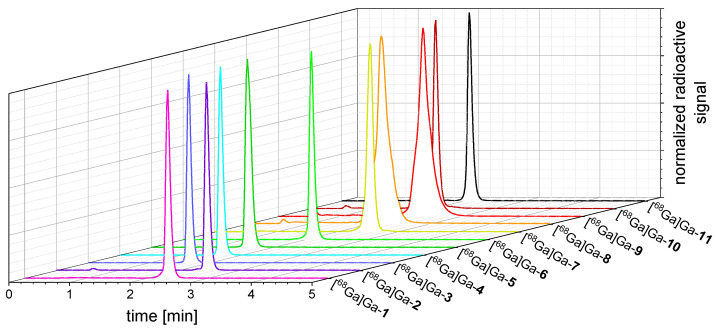
Analytical radio-HPLC chromatograms of [^68^Ga]Ga-**1**–[^68^Ga]Ga-**11** as obtained directly after radiolabeling of **1**–**11** with ^68^Ga^3+^.

The obtained chelator-modified labeling precursors were in the following radiolabeled with ^68^Ga^3+^, obtained in form of [^68^Ga]GaCl_3_ by fractioned elution of a commercially available ^68^Ge/^68^Ga-generator system. The pH of the generator eluate was, for this purpose, adjusted to 3.5–4.0 with sodium acetate solution (1.25 M, pH 4.6) before 1–20 nmol of the respective labeling precursor **1**–**11** were added. The reactions were conducted for 10 min at 45 °C, after which the radiometal incorporation was complete as determined by analytical radio-HPLC (Figure 4). The radiolabeled products [^68^Ga]Ga-**1**–[^68^Ga]Ga-**11** were obtained in non-optimized molar activities of 28–114 GBq/µmol.

The analytical radio-HPLC chromatograms demonstrate for [^68^Ga]Ga-**1**–[^68^Ga]Ga-**7**, [^68^Ga]Ga-**10** and [^68^Ga]Ga-**11** the expected high radiochemical purity of the obtained products, while for [^68^Ga]Ga-**8** and [^68^Ga]Ga-**9** very heterogeneous labeling products were obtained. The reason for this observation is not clear, as precursors **8** and **9** both showed (despite peak tailing in case of **9**) a high chemical purity under the same analytical HPLC conditions (Figure S1), thus excluding precursor impurities as a reason for the differently labeled products. A variation of the labeling conditions did not lead to a homogenization of the products either. 

Since both [^68^Ga]Ga-**8** and [^68^Ga]Ga-**9** showed high chemical stability as well as stability towards degradation by peptidases, the observed behavior might be due to the complexity of the peptidic precursors themselves, leading to an unexpected interaction of the radiometal ion with different structures of the peptides, presenting as different peaks during HPLC analysis. Interestingly, and despite the fact that radiometal-labeled hEGF derivatives have already been described, no analytical radio-HPLC data are available for such agents in the literature using a conventional C18-based material for HPLC analysis. The only example found of an analytical radio-HPLC chromatogram of such a tracer depicts the analysis of [^111^In]In-DTPA-hEGF using a size-exclusion chromatography system, also showing different peaks, implying an inhomogeneous product formed [43]. Altogether, this indicates that the formation of inhomogeneous products might be a common phenomenon for radiometal-labeled hEGF derivatives.

Although the observed product inhomogeneity obviously makes further evaluation of [^68^Ga]Ga-**8** and [^68^Ga]Ga-**9** difficult (since it is not known how and to what extent the individual species differ from each other), both agents were nevertheless further investigated together with [^68^Ga]Ga-**1**–[^68^Ga]Ga-**7**, [^68^Ga]Ga-**10** and [^68^Ga]Ga-**11** for the sake of completeness, even though their usefulness for in vivo EGFR targeting applications would be of limited value in the present form. 

For peptidic radiotracers, hydrophilicity is an important parameter as it can enable the initial assessment of the route by which the radioligand is metabolized and excreted [44,45]. The commonly determined measure for the hydrophilicity/lipophilicity profile of a radiotracer is the determination of its log*_D_*_(7.4)_ value and is investigated by partition experiments between an aqueous phosphate buffer phase at pH 7.4 and 1-octanol. For [^68^Ga]Ga-**1**–[^68^Ga]Ga-**11**, log*_D_*_(7.4)_ values of −3.44 ± 0.08 ([^68^Ga]Ga-**1**), −3.51 ± 0.22 ([^68^Ga]Ga-**2**), −3.92 ± 0.09 ([^68^Ga]Ga-**3**), −4.01 ± 0.13 ([^68^Ga]Ga-**4**), −3.91 ± 0.06 ([^68^Ga]Ga-**5**), −3.86 ± 0.11 ([^68^Ga]Ga-**6**), −3.03 ± 0.20 ([^68^Ga]Ga-**7**), −3.16 ± 0.11 ([^68^Ga]Ga-**8**), −2.93 ± 0.08 ([^68^Ga]Ga-**9**), −3.92 ± 0.09 ([^68^Ga]Ga-**10**) and −3.98 ± 0.06 ([^68^Ga]Ga-**11**) were obtained in these partition experiments. This high hydrophilicity of the tracers can be considered beneficial, as it should result in low, non-specific liver accumulation of the agents. This is advantageous with regard to the visualization of malignant liver lesions (especially hepatocellular carcinomas (HCCs) and liver metastases of colon carcinoma), which mostly overexpress the EGFR [46,47] and, thus, represent an important area of application of an EGFR-specific peptidic radiotracer.

For the applicability of radiopeptides, their stability towards degradation by human serum peptidases is of importance as a low stability towards metabolization interferes with successful target visualization. Thus, the stability of the radiopeptides [^68^Ga]Ga-**1**–[^68^Ga]Ga-**11** against degradation was determined by incubation with commercially available pooled human serum of healthy donors at 37 °C for 0, 15, 30, 45, 60, 75 and 90 min. At these time points, the fraction of the intact radiopeptides was again determined by analytical radio-HPLC, and the serum half-life of each radioligand was calculated. The results of these experiments are given in Figures S2–S12 and Table 1.

The results of the serum stability tests of [^68^Ga]Ga-**1**–[^68^Ga]Ga-**11** show that the peptides exhibit quite diverse stabilities towards degradation. While [^68^Ga]Ga-**7** shows a high susceptibility to the peptidases contained in the serum with a half-life of only 66.4 ± 3.0 min, [^68^Ga]Ga-**1**, [^68^Ga]Ga-**11** and [^68^Ga]Ga-**10** show markedly higher stabilities with half-lives of 295.7 ± 2.7, 562.0 ± 6.9 and 702.2 ± 42.2 min, respectively. For [^68^Ga]Ga-**2**–[^68^Ga]Ga-**6**, only a negligible degradation was observed, resulting in >1.000 min half-lives in serum. Thus, [^68^Ga]Ga-**1**–[^68^Ga]Ga-**6**, [^68^Ga]Ga-**10** and [^68^Ga]Ga-**11** show a sufficient stability whereas [^68^Ga]Ga-**7** exhibits most probably too high of a susceptibility to be a useful scaffold for the development of a tumor imaging agent based on it.

### 2.3. In Vitro Evaluation of [^68^Ga]Ga-***1***–[^68^Ga]Ga-***11*** Regarding Cell Uptake into A431 Cells and Receptor Affinity to the EGFR by Competitive Displacement Assays on the Same Cell Line

The radiolabeled agents [^68^Ga]Ga-**1**–[^68^Ga]Ga-**11** were in the following evaluated in terms of in vitro cell uptake into EGFR-positive A431 cells. The human epidermoid carcinoma A431 cell line is the standard cell system for the investigation of an EGFR-specificity of newly developed radiotracers [48,49], as it is known to highly overexpress the EGFR [50]. Furthermore, the EGFR-affinity of **1**–**11** was also determined on the same cell line by competitive displacement assays using [^125^I]I-hEGF as the competitor.

**Figure 5:**
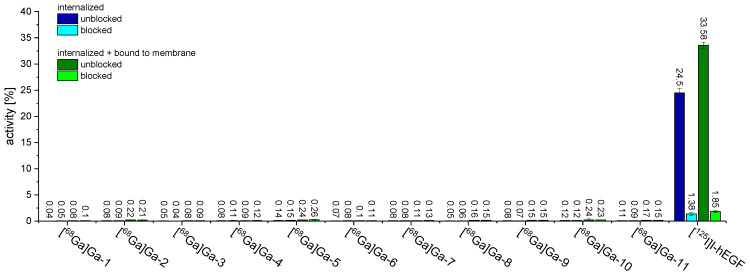
Summary of the results of the cell uptake studies of [^68^Ga]Ga-**1**–[^68^Ga]Ga-**11** and [^125^I]I-hEGF on EGFR-positive A431 cells after 1 h of incubation under non-blocking and blocking conditions. Each experiment was performed thrice, each in triplicate.

To quantify the receptor-specific tumor cell uptake, 10^6^ A431 cells were incubated with 50 pmol of [^68^Ga]Ga-**1**–[^68^Ga]Ga-**11** in the absence or presence of hEGF (human epidermal growth factor, 100-fold excess, 5 nmol) for blocking. The cellular uptake was differentiated by membrane-bound and internalized activity. In these experiments, no EGFR-specific interaction of any of the radiolabeled agents [^68^Ga]Ga-**1**–[^68^Ga]Ga-**11** was observed (Figure 5). 

To confirm that the methodology nevertheless works in principle, we also tested [^125^I]I-hEGF as reference compound under identical conditions and found—as expected—a substantial overall cellular uptake of 33.6% after 1 h of incubation, which was reduced to 1.9% under blocking conditions (Figure 5). 

Therefore, the methodology itself is suitable, indicating that none of the radioligands [^68^Ga]Ga-**1**–[^68^Ga]Ga-**11** show an EGFR-mediated tumor cell uptake or an associated EGFR-specific interaction. This result is at least in part surprising, since all peptides which the radiotracers developed here were based on have already been shown to interact with the EGFR. Although in the vast majority of descriptions, no receptor affinities were determined and an uptake into EGFR-positive cells was only shown qualitatively and not quantitatively, the complete absence of any EGFR-specific tumor cell uptake in all cases was not expected.

**Figure 6:**
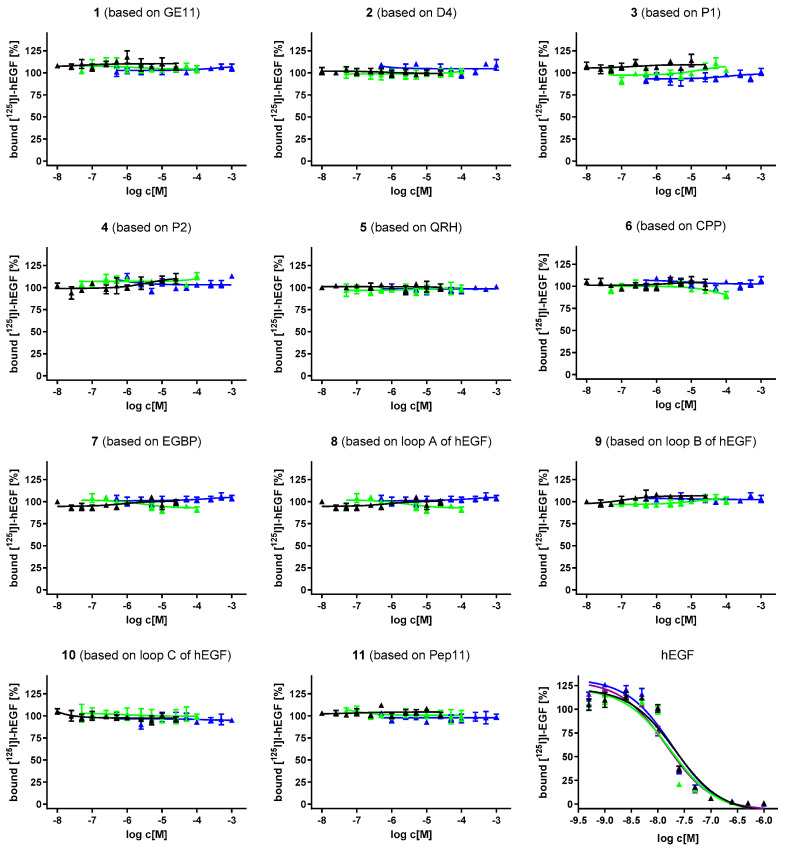
Graphical depiction of the results of the competitive displacements assays of **1**–**11** as well as the positive control hEGF on EGFR-positive A431 cells using [^125^I]I-hEGF as the competitor. Each experiment was performed at least thrice, each in triplicate, depicted in different colors.

To verify the results of the cell uptake studies, we further performed competitive displacement experiments for **1**–**11** on A431 cells by which IC_50_ values are obtained, serving as a measure of the affinity of the ligands to the EGFR. In these experiments, hEGF was used as the internal reference compound with known high affinity to the EGFR [51]. The binding curves obtained for **1**–**11** by these assays are depicted in Figure 6.

The data demonstrate that the reference peptide hEGF showed the expected high affinity to the EGFR target with an IC_50_ value of 15.2 ± 3.3 nM, being in the range of published values [51]. In contrast, none of the other agents were able to demonstrate an EGFR-specific binding and affinity to the target in a tested concentration up to 1 mM. As mentioned before, this was not expectable, as at least some EGFR-specific target interaction was postulated for the peptides GE11, D4, P1, P2, CPP, QRH, EGBP and Pep11. 

However, the fact that no receptor affinities have been published for almost all of the peptides to date and that only qualitative cell uptake studies have been described so far, in which the uptake was then usually only partially blockable, suggests that the suitability of the compounds for EGFR-targeting may be limited. This is confirmed by the evaluations performed here, which clearly show that none of the peptides GE11, D4, P1, P2, CPP, QRH, EGBP or Pep11 are suitable as a scaffold structure for future developments of radiolabeled compounds for targeting of EGFR-positive lesions.

Unfortunately, the truncated derivatives of hEGF also did not show the desired EGFR-specific interaction. On the contrary, the results clearly indicate that all three truncated variants based on the single loops of hEGF lost any EGFR specificity.

In view of the results obtained, we decided to not perform in vivo imaging and ex vivo biodistribution studies in A431 tumor-bearing xenograft animals, as it has been previously shown that tumor cell uptake [52] and affinity [53] directly correlate with in vivo tumor uptake. Thus, it must be expected that with using the radioligands developed here, no tumor uptake would be observed, not only in vitro but also under in vivo conditions. This assumption is supported by a very recent study where we developed an EGFR- and integrin α_v_β_3_-bispecific radioligand that showed no EGFR-associated A431 tumor cell uptake and affinity but only integrin-mediated U87MG tumor cell interaction in in vitro studies. A subsequent in vivo PET/CT imaging and ex vivo biodistribution study in A431 tumor-bearing xenograft mice—having been conducted to identify a potential EGFR-specific tumor uptake occurring despite the discouraging in vitro results—confirmed the absent EGFR-specificity of the developed agent not only in vitro, but also in vivo [54].

Thus, no peptide could be identified in this study that is suitable as a scaffold structure for the future development of EGFR-specific PET radiotracers based on it. To achieve this goal, it will first be necessary to find a suitable peptide structure that is capable of EGFR-specific binding with high affinity.

## 3. Materials and Methods

*General: Chemicals, radiochemicals and materials for in vitro assays.* All chemicals were purchased from commercial sources in analytical grade quality and used without further purification unless otherwise stated. Fmoc- and side chain-protected amino acids and all resins Fmoc-Ile-Wang resin LL (100–200 mesh) (loading 0.31–0.40 mmol/g), Fmoc-Thr(*^t^*Bu) Wang resin (loading 0.65 mmol/g), Rink Amide MBHA resin LL (100–200 mesh) (loading 0.36 mmol/g), Fmoc-Asn(Trt)-2-Cl-Trt resin (loading 1.1 mmol/g) as well as benzotriazol-1-yloxytripyrrolidinophosphonium hexafluorophosphate (PyBOP) were purchased from NovaBiochem (Darmstadt, Germany). 4-(4,7-*Bis*(2-(*t*-butoxy)-2-oxoethyl)-1,4,7-triazacyclononan-1-yl)-5-(tert-butoxy)-5-oxo-pentanoic acid ((*R*)-NODA-GA(*^t^*Bu)_3_) was purchased from CheMatech (Dijon, France). 21-(9-Fluorenylmethyloxycarbonyl)amino-4,7,10,13,16,19-hexaoxaheneicosanoic acid (PEG_5_, Fmoc-NH-PEG_5_-COOH) was obtained from Iris Biotech (Marktredwitz, Germany). Dichloromethane (DCM), diethylether, dimethylformamide (DMF), piperidine, 2-(1*H*-benzotriazol-1-yl)-1,1,3,3-tetramethyluronium hexafluorophosphate (HBTU), trifluoroacetic acid (TFA) and water were purchased from Carl Roth (Karlsruhe, Germany), acetonitrile (MeCN) from Häberle Labortechnik (Lonsee-Ettlenschieß, Germany), *N,N*-diisopropylethylamine (DIPEA), triisopropylsilane (TIS) and *N*-chlorosuccinimide (NCS) from Sigma Aldrich (Taufkirchen, Germany). Human serum (pooled serum from male AB clotted whole blood) was also purchased from Sigma Aldrich (Taufkirchen, Germany). A431 epidermoid carcinoma cells were obtained from DSMZ (Braunschweig, Germany) and cell culture media from Bio&SELL (Feucht, Germany). [^125^I]I-hEGF in a molar activity of 81.4 TBq/mmol was obtained from PerkinElmer as custom synthesis (NEX083000MC, Rodgau, Germany). ^68^GaCl_3_ for ^68^Ga-radiolabeling reactions was obtained from a GalliaPharm ^68^Ge/^68^Ga-generator system (Eckert&Ziegler, Berlin, Germany). H_2_O (Tracepur quality), hydrochloric acid (30%, Suprapur quality) and sodium hydroxide (30%, Suprapur quality) for radiolabeling reactions were purchased from Merck (Darmstadt, Germany).

*Instrumentation.* HPLC: Analytical HPLC, semipreparative HPLC and analytical radio-HPLC were conducted utilizing a Dionex UltiMate 3000 system together with Chromeleon Software (Version 6.80). For analytical chromatography, a Chromolith Performance (RP-18e, 100–4.6 mm, Merck, Germany) and for semipreparative analyses, Chromolith SemiPrep (RP-18e, 100–10 mm, Merck, Germany) columns were used, respectively. For radioanalytical chromatography, a Dionex UltiMate 3000 system equipped with a Raytest GABI* radioactivity detector was used together with a Chromolith Performance (RP-18e, 100–4.6 mm, Merck, Germany) column. All operations were performed with a flow rate of 4 mL/min using H_2_O supplemented with 0.1% TFA and MeCN also supplemented with 0.1% TFA as solvents. MALDI-TOF MS: Matrix-Assisted Laser Desorption/Ionization (MALDI) time-of-flight mass spectra were obtained utilizing a Bruker Daltonics Microflex spectrometer, linear acquisition mode, positive ion source and 200 shots per spot. α-Cyano-4-hydroxycinnamic acid (α-CS) was chosen as matrix, and the dried-droplet method was used for sample preparation on a micro scout target (MSP 96 target polished steel BC, Bruker Daltonics, Germany). The data were recorded with flexControl Version 3.3 and analyzed with flexAnalysis Version 3.3 software. HR-ESI-MS: For high-resolution electrospray ionization mass spectroscopy (HR-ESI-MS), a Thermo Finnigan LTQ FT Ultra Fourier Transform Ion Cyclotron Resonance (Dreieich, Germany) mass spectrometer was used. γ-Counting was performed using a 2480 Wizard^2^ gamma counter system from Perkin Elmer. Ultrasonic bath: Ultrasound-assisted syntheses were performed in a Bandelin Sonorex Super RK 225 H ultrasonic bath (Berlin, Germany) with the temperature of the water being kept at ambient temperature.

*Peptide Syntheses.* Solid-phase peptide synthesis (SPPS) was performed according to standard Fmoc protocols [37] in standard syringes (5 mL, HSW, Tuttlingen, Germany) being equipped with two layers of 35 μm porous high-density polyethylene frits (Reichelt Chemietechnik, Heidelberg, Germany). The resin was placed between plunger and frit, swollen for 45 min in DCM and washed with DMF directly before coupling of the first amino acid. Coupling reactions were carried out in DMF for 15 min in an ultrasonic bath at ambient temperature using 2 equiv. of the respective amino acid and 1.9 equiv. of HBTU as the coupling reagent with 2 equiv. of DIPEA as the base. Fmoc-protecting groups were removed using 50% piperidine in DMF (*v*/*v*) (2 + 5 min). The removal of acid-labile protecting groups and simultaneous cleavage from the resin was performed using a mixture of TFA, TIS and H_2_O (95/2.5/2.5, *v*/*v*/*v*) for 3 h at room temperature, followed by evaporation of the volatile materials. The residues were dissolved in 1:1 MeCN/H_2_O + 0.1% TFA (*v*/*v*), purified by semipreparative HPLC and subsequently lyophilized. 

### Syntheses of the Peptidic Labeling Precursors ***1***–***11***

*NODA-GA-PEG_5_-GE11 (***1***).* NODA-GA-PEG_5_-Tyr-His-Trp-Tyr-Gly-Tyr-Thr-Pro-Gln-Asn-Val-Ile (**1**) was synthesized on solid support according to standard amino acid coupling protocols on a commercially available Fmoc-Ile-Wang resin LL (100–200 mesh, loading 0.31–0.40 mmol/g), standard *N*_α_-Fmoc amino acids, HBTU as coupling reagent, Fmoc-NH-PEG_5_-COOH and (*R*)-NODA-GA(*^t^*Bu)_3_. Cleavage from the resin, removal of acid-labile protecting groups and purification was achieved as described. The obtained product was a colorless solid after lyophilization. Gradient for purification: 20–40% MeCN + 0.1% TFA in 8 min (R_t_ = 5.42 min), yield: 50%. MALDI-TOF-MS (*m/z*) using α-cyano-4-hydroxycinnamic acid as matrix substance for [M + H]^+^ (calculated): 2233.76 (2234.07). HR-ESI-MS (*m/z*) [M + 2H]^2+^ (calculated): 1117.5400 (1117.5403), [M-2H]^2−^ (calculated): 1115.5249 (1115.5257), [M-4H + 3K]^2−^ (calculated): 1172.5213 (1172.4618).

*NODA-GA-PEG_5_-D4 (***2***).* NODA-GA-PEG_5_-Leu-Ala-Arg-Leu-Leu-Thr (**2**) was synthesized on solid support according to standard amino acid coupling protocols on a commercially available Fmoc-Thr(*^t^*Bu)-Wang Harz (loading 0.65 mmol/g, 32.5 µmol), standard *N*_α_-Fmoc amino acids and Fmoc-NH-PEG_5_-COOH with HBTU as coupling reagent. (*R*)-NODA-GA(*^t^*Bu)_3_ was conjugated utilizing PyBOP instead of HBTU and prolonged reaction times of 30 min. Cleavage from the resin, removal of acid-labile protecting groups and purification was achieved as described. The obtained product was a colorless solid after lyophilization. Gradient for purification: 10–60% MeCN + 0.1% TFA in 8 min (R_t_ = 5.84 min), yield: 74%. MALDI-TOF-MS (*m/z*) using α-cyano-4-hydroxycinnamic acid as matrix substance for [M + H]^+^ (calculated): 1378.51 (1378.80). HR-ESI-MS (*m/z*) [M + 2H]^2+^ (calculated): 689.9068 (689.9056), [M + H]^+^ (calculated): 1378.8104 (1378.8040) [M-H]^−^ (calculated): 1376.7881 (1376.7893).

*NODA-GA-PEG_5_-P1 (***3***).* NODA-GA-PEG_5_-Ser-Tyr-Pro-Ile-Pro-Asp-Thr (**3**) was synthesized on solid support according to standard amino acid coupling protocols on a commercially available Fmoc-Thr(*^t^*Bu) Wang resin (loading 0.3 mmol/g, 30.3 µmol), standard *N*_α_-Fmoc amino acids and Fmoc-NH-PEG_5_-COOH with HBTU as coupling reagent. (*R*)-NODA-GA(*^t^*Bu)_3_ was conjugated utilizing PyBOP instead of HBTU and prolonged reaction times of 30 min. Cleavage from the resin, removal of acid-labile protecting groups and purification was achieved as described. The product was obtained as a colorless solid after lyophilization. Gradient for purification: 10–60% MeCN + 0.1% TFA in 8 min (R_t_ = 4.37 min), yield: 59%. MALDI-TOF-MS (*m/z*) using α-cyano-4-hydroxycinnamic acid as matrix substance for [M + H]^+^ (calculated): 1484.41 (1484.73). HR-ESI-MS (*m/z*) [M + H]^+^ (calculated): 1484.7262 (1484.7255), [M-H]^−^ (calculated): 1482.7088 (1482.7108), [M-2H]^2−^ (calculated): 740.8505 (740.8518), [M-3H]^3−^ (calculated): 493.5652 (493.5654).

*NODA-GA-PEG_5_-P2 (***4***).* NODA-GA-PEG_5_-His-Thr-Ser-Asp-Gln-Thr-Asn (**4**) was synthesized on solid support according to standard amino acid coupling protocols on a commercially available Fmoc-Asn(Trt)-2-Cl-Trt resin (loading 1.1 mmol/g, 44 µmol), standard *N*_α_-Fmoc amino acids and Fmoc-NH-PEG_5_-COOH with HBTU as coupling reagent. (*R*)-NODA-GA(*^t^*Bu)_3_ was conjugated utilizing PyBOP instead of HBTU and prolonged reaction times of 30 min. Cleavage from the resin, removal of acid-labile protecting groups and purification was achieved as described. The product was obtained as a colorless solid after lyophilization. Gradient: 10–60% MeCN + 0.1% TFA in 8 min (R_t_ = 2.87 min), yield: 21%. MALDI-TOF-MS (*m/z*) using α-cyano-4-hydroxycinnamic acid as matrix substance for [M + H]^+^ (calculated): 1494.10 (1494.68). HR-ESI-MS (*m/z*) [M + 2H]^2+^ (calculated): 747.8441 (747.8440), [M + H]^+^ (calculated): 1494.6817 (1494.6806), [M-H]^−^ (calculated): 1492.6620 (1492.6660), [M-2H]^2−^ (calculated): 745.8290 (745.8294).

*QRH-PEG_5_-NODA-GA (***5***).* Gln-Arg-His-Lys-Pro-Arg-Glu-PEG_5_-Lys(NODA-GA)-NH_2_ (**5**) was synthesized on solid support according to standard amino acid coupling protocols on a commercially available Rink amide MBHA LL resin (loading 0.36 mmol/g, 27 µmol), standard *N*_α_-Fmoc amino acids, Fmoc-Lys(Mtt)-OH and Fmoc-NH-PEG_5_-COOH with HBTU as coupling reagent. After conjugation of Fmoc-NH-PEG_5_-COOH, the Mtt-protecting group of the lysine was removed with TFA in DCM (1/99, *v*/*v*) within 30–45 min, followed by threefold washing of the resin with first DCM and afterwards DIPEA in DCM (9/1, *v*/*v*). (*R*)-NODA-GA(*^t^*Bu)_3_ was conjugated to the *N*_ε_-amino functionality of the lysine utilizing PyBOP instead of HBTU and prolonged reaction times of 30 min. Afterwards, the other amino acid building blocks were coupled until the sequence was complete. Cleavage from the resin, removal of acid-labile protecting groups and purification was achieved as described. The obtained product was a colorless solid after lyophilization. Gradient: 10–60% MeCN + 0.1% TFA in 8 min (R_t_ = 3.03 min), yield: 50%. MALDI-TOF-MS (*m/z*) using α-cyano-4-hydroxycinnamic acid as matrix substance for [M + H]^+^ (calculated): 1769.95 (1769.99). HR-ESI-MS (*m/z*) [M + 3H]^3+^ (calculated): 590.6674 (590.6671), [M + 2H]^2+^ (calculated): 885.4981 (885.4971), [M-H]^−^ (calculated): 1767.9665 (1767.9722).

*CPP-PEG_5_-NODA-GA (***6***).* Asn-Arg-Pro-Asp-Ser-Ala-Gln-Phe-Trp-Leu-His-His-PEG_5_-Lys(NODA-GA)-NH_2_ (**6**) was synthesized on solid support according to standard amino acid coupling protocols on a commercially available Rink amide MBHA LL resin (loading 0.36 mmol/g, 36 µmol), standard *N*_α_-Fmoc amino acids, Fmoc-Lys(Mtt)-OH and Fmoc-NH-PEG_5_-COOH with HBTU as coupling reagent. After conjugation of Fmoc-NH-PEG_5_-COOH, the Mtt-protecting group of the lysine was removed with TFA in DCM (1/99, *v*/*v*) within 30–45 min, followed by threefold washing of the resin with first DCM and afterwards DIPEA in DCM (9/1, *v*/*v*). (*R*)-NODA-GA(*^t^*Bu)_3_ was conjugated to the *N*_ε_-amino functionality of the lysine utilizing PyBOP instead of HBTU and prolonged reaction times of 30 min. Afterwards, the other amino acid building blocks were coupled until the sequence was complete. Cleavage from the resin, removal of acid-labile protecting groups and purification was achieved as described. The product was obtained as a colorless solid after lyophilization. Gradient: 10–60% MeCN + 0.1% TFA in 8 min (R_t_ = 4.55 min), yield: 49%. MALDI-TOF-MS (*m/z*) using α-cyano-4-hydroxycinnamic acid as matrix substance for [M + H]^+^ (calculated): 2327.20 (2328.18). HR-ESI-MS (*m/z*) [M + 4H]^4+^ (calculated): 582.8006 (582.8008), [M + 3H]^3+^ (calculated): 776.7317 (776.7319), [M + 2H]^2+^ (calculated): 1164.5946 (1164.5943).

*EGBP-PEG_5_-NODA-GA (***7***).* Phe-Pro-Met-Phe-Asn-His-Trp-Glu-Gln-Trp-Pro-Pro-PEG_5_-Lys(NODA-GA)-NH_2_ (**7**) was synthesized on solid support according to standard amino acid coupling protocols on a commercially available Rink amide MBHA LL resin (loading 0.36 mmol/g, 36 µmol), standard *N*_α_-Fmoc amino acids, Fmoc-Lys(Mtt)-OH and Fmoc-NH-PEG_5_-COOH with HBTU as coupling reagent. After conjugation of Fmoc-NH-PEG_5_-COOH, the Mtt-protecting group of the lysine was removed with TFA in DCM (1/99, *v*/*v*) within 30–45 min, followed by threefold washing of the resin with first DCM and afterwards DIPEA in DCM (9/1, *v*/*v*). (*R*)-NODA-GA(*^t^*Bu)_3_ was conjugated to the *N*_ε_-amino functionality of the lysine utilizing PyBOP instead of HBTU and prolonged reaction times of 30 min. Afterwards, the other amino acid building blocks were coupled until the sequence was complete. Cleavage from the resin, removal of the acid-labile protecting groups and purification was achieved as described. The obtained product was a colorless solid after lyophilization. Gradient: 10–60% MeCN + 0.1% TFA in 8 min (R_t_ = 5.78 min), yield: 32%. MALDI-TOF-MS (*m/z*) using α-cyano-4-hydroxycinnamic acid as matrix substance for [M + H]^+^ (calculated): 2435.47 (2436.78). HR-ESI-MS (*m/z*) [M + 3H]^3+^ (calculated): 812.7310 (812.7307), [M + 2H]^2+^ (calculated): 1218.5933 (1218.5924), [M + H]+ (calculated): (2436.1774), [M-2H]^2−^ (calculated): 1216.5715 (1216.5778).

*EGF_5-21_-PEG_5_-NODA-GA (***8***).* Glu-c[Cys-Pro-Leu-Ser-His-Asp-Gly-Tyr-Cys-Leu-His-Asp-Gly-Val-Cys]-Met-PEG_5_-Lys(NODA-GA)-NH_2_ (**8**) was synthesized on solid support according to standard amino acid coupling protocols on a commercially available Rink amide MBHA LL resin (loading 0.36 mmol/g, 27 µmol), standard *N*_α_-Fmoc amino acids, Fmoc-Lys(Mtt)-OH, Fmoc-Cys(Mmt)-OH and Fmoc-NH-PEG_5_-COOH with HBTU as coupling reagent. After conjugation of Fmoc-NH-PEG_5_-COOH, the Mtt-protecting group of the lysine was removed with TFA in DCM (1/99, *v*/*v*) within 30–45 min, followed by threefold washing of the resin with first DCM and afterwards DIPEA in DCM (9/1, *v*/*v*). (*R*)-NODA-GA(*^t^*Bu)_3_ was conjugated to the *N*_ε_-amino functionality of the lysine utilizing PyBOP instead of HBTU and prolonged reaction times of 30 min. Afterwards, the other amino acid building blocks were coupled until the sequence was complete. Before cleaving the peptide from the resin, the Mmt-protecting group of the cysteines (EGF_6_ and EGF_20_) were removed with TFA in DCM (2/98, *v*/*v*) within 45 min, followed by threefold washing of the resin with first DCM and afterwards DIPEA in DCM (9/1, *v*/*v*). The disulfide bond was formed by oxidation of the two thiols utilizing *N*-chlorosuccinimide (NCS) (1.05 equiv.) for 15 min at ambient temperature. Cleavage from the resin, removal of acid-labile protecting groups and purification was achieved as described. The obtained product was a colorless solid after lyophilization. Gradient: 0–100% MeCN + 0.1% TFA in 8 min (R_t_ = 4.54 min), yield: 22%. MALDI-TOF-MS (*m/z*) using α-cyano-4-hydroxycinnamic acid as matrix substance for [M + H]^+^ (calculated): 2697.48 (2697.17). HR-ESI-MS (*m/z*) [M + 3H]^3+^ (calculated): 899.7312 (899.7297), [M + 2H]^2+^ (calculated): 1349.0952 (1349.0909), [M-2H]^2−^ (calculated): 1347.0750 (1347.0763).

*EGF_13-32_-PEG_5_-NODA-GA (***9***).* Tyr-c[Cys-Leu-His-Aps-Gly-Val-Cys-Met-Tyr-Ile-Glu-Ala-Leu-Asp-Lys-Tyr-Ala-Cys]-Asn-PEG_5_-Lys(NODA-GA)-NH_2_ (**9**) was synthesized on solid support according to standard amino acid coupling protocols on a commercially available Rink amide MBHA LL resin (loading 0.36 mmol/g, 27 µmol), standard *N*_α_-Fmoc amino acids, Fmoc-Lys(Mtt)-OH, Fmoc-Cys(Mmt)-OH and Fmoc-NH-PEG_5_-COOH with HBTU as coupling reagent. After conjugation of Fmoc-NH-PEG_5_-COOH, the Mtt-protecting group of the lysine was removed with TFA in DCM (1/99, *v*/*v*) within 30–45 min, followed by threefold washing of the resin with first DCM and afterwards DIPEA in DCM (9/1, *v*/*v*). (*R*)-NODA-GA(*^t^*Bu)_3_ was conjugated to the *N*_ε_-amino functionality of the lysine utilizing PyBOP instead of HBTU and prolonged reaction times of 30 min. Afterwards, the other amino acid building blocks were coupled until the sequence was complete. Before cleaving the peptide from the resin, the Mmt-protecting group of the cysteines (EGF_14_ and EGF_31_) were removed with TFA in DCM (2/98, *v*/*v*) within 45 min, followed by threefold washing of the resin with first DCM and afterwards DIPEA in DCM (9/1, *v*/*v*). The disulfide bond was formed by oxidation of the two thiols utilizing *N*-chlorosuccinimide (NCS) (1.05 equiv.) for 15 min at ambient temperature. Cleavage from the resin, removal of acid-labile protecting groups and purification was achieved as described. The obtained product was a colorless solid after lyophilization. Gradient: 10–60% MeCN + 0.1% TFA in 8 min (R_t_ = 5.49 min), yield: 5%. MALDI-TOF-MS (*m/z*) using α-cyano-4-hydroxycinnamic acid as matrix substance for [M + H]^+^ (calculated): 3127.48 (3126.46). HR-ESI-MS (*m/z*) [M + 4H]^4+^ (calculated): 782.3710 (782.3692), [M + 3H]^3+^ (calculated): 1042.8266 (1042.8232).

*EGF_32-43_-PEG_5_-NODA-GA (***10***).* Asn-c[Cys-Val-Val-Gly-Tyr-Ile-Gly-Glu-Arg-Cys]-Gln-PEG_5_-Lys(NODA-GA)-NH_2_ (**10**) was synthesized on solid support according to standard amino acid coupling protocols on a commercially available Rink amide MBHA LL resin (loading 0.36 mmol/g, 27 µmol), standard *N*_α_-Fmoc amino acids, Fmoc-Lys(Mtt)-OH, Fmoc-Cys(Mmt)-OH and Fmoc-NH-PEG_5_-COOH with HBTU as coupling reagent. After conjugation of Fmoc-NH-PEG_5_-COOH, the Mtt-protecting group of the lysine was removed with TFA in DCM (1/99, *v*/*v*) within 30–45 min, followed by threefold washing of the resin with first DCM and afterwards DIPEA in DCM (9/1, *v*/*v*). (*R*)-NODA-GA(*^t^*Bu)_3_ was conjugated to the *N*_ε_-amino functionality of the lysine utilizing PyBOP instead of HBTU and prolonged reaction times of 30 min. Afterwards, the other amino acid building blocks were coupled until the sequence was complete. Before cleaving the peptide from the resin, the Mmt-protecting group of the cysteines (EGF_33_ and EGF_42_) were removed with TFA in DCM (2/98, *v*/*v*) within 45 min, followed by threefold washing of the resin with first DCM and afterwards DIPEA in DCM (9/1, *v*/*v*). The disulfide bond was formed by oxidation of the two thiols utilizing *N*-chlorosuccinimide (NCS) (1.05 equiv.) for 15 min at ambient temperature. Cleavage from the resin, removal of acid-labile protecting groups and purification was achieved as described. The product was obtained as a colorless solid after lyophilization. Gradient: 10–60% MeCN + 0.1% TFA in 8 min (R_t_ = 4.47 min), yield: 10%. MALDI-TOF-MS (*m/z*) using α-cyano-4-hydroxycinnamic acid as matrix substance for [M + H]^+^ (calculated): 2158.12 (2158.05). HR-ESI-MS (*m/z*) [M + 2H]^2+^ (calculated): 1080.0339 (1080.0313), [M + 2H]^2−^ (calculated): 1078.0131 (1078.0167).

*Pep11-PEG_5_-NODA-GA (***11***).* Trp-Ser-Gly-Glu-Asn-Gly-Pro-Gly-Phe-Tyr-Asp-Tyr-Glu-Ala-PEG_5_-Lys(NODA-GA)-NH_2_ (**11**) was synthesized on solid support according to standard amino acid coupling protocols on a commercially available Rink amide MBHA LL resin (loading 0.36 mmol/g, 36 µmol), standard *N*_α_-Fmoc amino acids, Fmoc-Lys(Mtt)-OH and Fmoc-NH-PEG_5_-COOH with HBTU as coupling reagent. After conjugation of Fmoc-NH-PEG_5_-COOH, the Mtt-protecting group of the lysine was removed with TFA in DCM (1/99, *v*/*v*) within 30–45 min, followed by threefold washing of the resin with first DCM and afterwards DIPEA in DCM (9/1, *v*/*v*). (*R*)-NODA-GA(*^t^*Bu)_3_ was conjugated to the *N*_ε_-amino functionality of the lysine utilizing PyBOP instead of HBTU and prolonged reaction times of 30 min. Afterwards, the other amino acid building blocks were coupled until the sequence was complete. Cleavage from the resin, removal of acid-labile protecting groups and purification was achieved as described. The obtained product was a colorless solid after lyophilization. Gradient: 10–40% MeCN + 0.1% TFA in 8 min (R_t_ = 5.99 min), yield: 54%. MALDI-TOF-MS (*m/z*) using α-cyano-4-hydroxycinnamic acid as matrix substance for [M + H]^+^ (calculated): 2412.34 (2412.10). HR-ESI-MS (*m/z*) [M + 2H]^2+^ (calculated): 1206.5559 (1206.5516), [M-2H]^2−^ (calculated): 1204.5350 (1204.5370), [M-3H]^3−^ (calculated): 802.6877 (802.6889).

*^68^Ga-Radiolabeling of ***1***–***11***.* For radiolabeling of **1**–**11** with ^68^Ga^3 +^, [^68^Ga]GaCl_3_ was obtained by fractioned elution of a commercial ^68^Ge/^68^Ga-generator system (GalliaPharm, Eckert & Ziegler) with 0.1 M hydrochloric acid (HCl). The peptides (1–20 nmol) in H_2_O (Tracepur quality) were added to 50–250 MBq [^68^Ga]GaCl_3_ (0.5–1 mL) and the pH was adjusted to 3.5–4.0 by adding sodium acetate solution (pH 4.6, 1.25 M, 100–250 µL). After 10 min incubation at 45 °C, the radiochemical purity was determined by analytical radio-HPLC. The radiolabeled products [^68^Ga]Ga-**1**–[^68^Ga]Ga-**7**, [^68^Ga]Ga-**10** and [^68^Ga]Ga-**11** were found to be 98–100% pure and obtained in non-optimized molar activities of 28–114 GBq/µmol. 

*Determination of the log_D(7_._4)_ values of [^68^Ga]Ga-***1***–[^68^Ga]Ga-***11***.* The aqueous phase_pH7_._4_/1-octanol partition coefficient (log*_D_*_(7.4)_) of the developed radiotracers was determined by addition of a solution of the respective radiolabeled peptide (5 µL, 0.5–2 MBq) to a mixture of 1-octanol (800 µL) and phosphate buffer (0.05 M, pH 7.4, 795 µL). The mixtures were vigorously shaken for 5 min on a mechanical shaker and subsequently centrifuged at 13,000 rpm (12,100× *g*) for 2 min to achieve complete phase separation. 125 µL of each phase were taken and the amount of radioactivity in each phase was determined by γ-counting. Experiments were performed thrice, each in triplicate. 

*Determination of the stability of [^68^Ga]Ga-***1***–[^68^Ga]Ga-***11*** towards degradation by human serum peptidases.* A sample of the respective radiolabeled peptide (125 µL, 10–30 MBq, pH 7.4) was added to commercially available pooled human serum (500 µL) and warmed to 37 °C. At defined time-points (t = 0, 5, 15, 30, 45, 60 and 90 min), 75 µL of the mixture were added to 75 µL ice-cold ethanol, further cooled on ice for 5 min, and centrifuged at 13,000 rpm (12,100× *g*) for 90 s. The supernatant was collected, the activity of the supernatant and the precipitate measured, where after the supernatant was analyzed by analytical radio-HPLC and the amount of intact tracer was quantified. Experiments were performed thrice for each radioligand.

*Cell culture.* A431 human epidermoid carcinoma cells were grown in DMEM, high glucose medium (Gibco^TM^, FisherScientific, Schwerte, Germany) supplemented with fetal bovine serum (FBS) and penicillin-streptomycin (10,000 U/mL) (Gibco^TM,^ FisherScientific, Schwerte, Germany) (89/10/1, *v*/*v*/*v*) at 37 °C in a humidified CO_2_ (5%) atmosphere and were split at >80% confluence. 

*Internalization studies of [^68^Ga]Ga-***1***–[^68^Ga]Ga-***11*** on A431 cells.* For this purpose, the A431 cells (10^6^ per well) were seeded into 24-well cell culture multiwell plates (cellstar) and incubated for 2 days at 37 °C in a humidified CO_2_ (5%) atmosphere. Directly before the experiment, each well was washed twice with fresh internalization medium (PBS/DMEM, high glucose, 1/99, *v*/*v*), followed by the addition of internalization medium to a final volume of 1.35 mL per well. To half of the wells, BSA/PBS (150 µL, 0.5/99.5, *w*/*v)* was added, while to the other half, a blocking solution of a 100-fold molar excess of hEGF (5 nM) in BSA/PBS (150 µL, 0.5/99.5, *w*/*v)* was added. After incubation at 37 °C for 10 min, a solution of the respective radiolabeled peptide (0.05 nM) in internalization medium (8 µL) was added to each well and the plate was incubated for another 1 h. Afterwards, the medium was separated from the cells and each well was washed twice with 1 mL of ice-cold internalization medium, the washing solutions were added to the collected internalization medium and measured in a γ-counter (these combined solutions contain the unbound fraction). To determine the surface-bound fraction, each well was further incubated twice for 5 min with ice-cold glycine-buffer (pH 2.8, 0.05 M, 1 mL) and the supernatants were collected and measured. The internalized fraction was determined by lysing the cells twice for 5 min with sodium hydroxide solution (2 M, 1 mL), collection and measurement of the combined solutions. The amount of radiotracer in each fraction was determined by γ-counting and referenced against a standard solution of the radiolabeled peptide (8 µL). Experiments were performed thrice, each in triplicate. 

*Competitive displacement studies of [^68^Ga]Ga-***1***–[^68^Ga]Ga-***11*** on A431 cells.* MultiScreen_HTS_-BV, 1,2 µm 96 well plates were conditioned for one hour prior to the respective experiment with BSA/PBS (1/99, *w*/*v,* 200 µL per well) solution before use. A431 cells (10^5^ per well), suspended in Opti-MEM I (GlutaMAX I) medium (50 µL), were seeded and the plate was incubated at 37 °C for 1 h with 0.25 kBq [^125^I]I-hEGF (25 µL) in the presence of eleven increasing concentrations ranging from 10^−8^ M to 10^−3^ M of the respective competitor (**1**–**11**, 25 µL) or 5 × 10^−10^ M to 10^−6^ M (hEGF, 25 µL), respectively, keeping one well empty ensuring 100% binding of the radioligand. After the incubation, the filters were washed three times with ice-cold PBS (1 × 200 μL, 2 × 100 μL) to remove unbound [^125^I]I-hEGF, collected and measured in a γ-counter. The 50% inhibitory concentration (IC_50_) values for each compound were obtained by nonlinear regression analysis using GraphPad Prism Software (v5.04). Each experiment was performed at least three times, in triplicate.

## 4. Conclusions

Although eight different already known peptides (GE11, D4, P1, P2, CPP, QRH, EGBP and Pep11) as well as three truncated derivatives of hEGF based on the three loops of the peptide were investigated in this study with regard to their suitability as a basis for the development of EGFR-specific radiopharmaceuticals, no relevant EGFR-specific interaction could be demonstrated for any of the assessed compounds.

Thus, to replace protein-based radiotracers for the visualization of EGFR-positive tumor lesions by a radiopeptide, the next step is to develop or identify a suitable peptidic receptor ligand that exhibits a sufficiently strong EGFR interaction profile.

## Figures and Tables

**Table 1:** Summary of the results of the log*_D_*_(7_._4)_ determination experiments and serum stability tests for [^68^Ga]Ga-**1**–[^68^Ga]Ga-**11**.

Compound	log*_D_*_(7.4)_	Calculated Half-Life in Human Serum [min]
[^68^Ga]Ga-**1**	−3.44 ± 0.08	295.7 ± 2.7
[^68^Ga]Ga-**2**	−3.51 ± 0.22	2089.2 ± 52.3
[^68^Ga]Ga-**3**	−3.92 ± 0.09	8077.7 ± 307.2
[^68^Ga]Ga-**4**	−4.01 ± 0.13	2427.2 ± 38.7
[^68^Ga]Ga-**5**	−3.91 ± 0.06	– ^1^
[^68^Ga]Ga-**6**	−3.86 ± 0.11	1064.1 ± 43.0
[^68^Ga]Ga-**7**	−3.03 ± 0.20	66.4 ± 3.0
[^68^Ga]Ga-**8**	−3.16 ± 0.11	–
[^68^Ga]Ga-**9**	−2.93 ± 0.08	–
[^68^Ga]Ga-**10**	−3.92 ± 0.09	702.2 ± 42.2
[^68^Ga]Ga-**11**	−3.98 ± 0.06	562.0 ± 6.9

^1^ Half-life in human serum could not be determined as the agent showed no detectable degradation by serum peptidases.

## Data Availability

Data is contained within the article and supplementary material.

## Supplementary Materials

The following supporting information can be downloaded at: https://www.mdpi.com/article/10.3390/ph16020273/s1, Figure S1: Analytical HPLC chromatograms of **1**–**11** after purification; Figures S2–S12: Results of the determination of the stability of [^68^Ga]Ga-**1**–[^68^Ga]Ga-**11** in human serum.
