# Supplementary material for: Towards Radiolabeled EGFR-Specific Peptides: Alternatives to GE11
Source: Pharmaceuticals (Basel). 2023 Feb 11;16(2):273. doi: 10.3390/ph16020273 (PMC9963887; doi:10.3390/ph16020273)
Supplement: Supplementary file 1 [file pharmaceuticals-16-00273-s001.zip › pharmaceuticals-2211515-supplementary.pdf]

Towards radiolabeled EGFR-specific peptides: Alternatives to GE11

Benedikt Judmann,[1,2] Björn Wängler,[2] Ralf Schirrmacher,[3] Gert Fricker,[4] Carmen Wängler[1*]

[1] Biomedical Chemistry, Clinic of Radiology and Nuclear Medicine, Medical Faculty Mannheim, Heidelberg University, 68167 Mannheim, Germany
[2] Molecular Imaging and Radiochemistry, Clinic of Radiology and Nuclear Medicine, Medical Faculty Mannheim, Heidelberg University, 68167 Mannheim, Germany
[3] Department of Oncology, Division of Oncological Imaging, University of Alberta, Edmonton, AB T6G 1Z2, Canada
[4] Institute of Pharmacy and Molecular Biotechnology, University of Heidelberg, 69120 Heidelberg, Germany

Content

Analytical HPLC chromatograms of **1** – **11**	page S2
Results of the determination of the stability of [^{68}Ga]Ga-**1** – [^{68}Ga]Ga-**11** in human serum (analytical radio-HPLC chromatograms of the tracers incubated for 0, 15, 30, 45, 60, 75 and 90 minutes with human serum)	pages S2 – S7

Analytical HPLC chromatograms of **1 – 11**

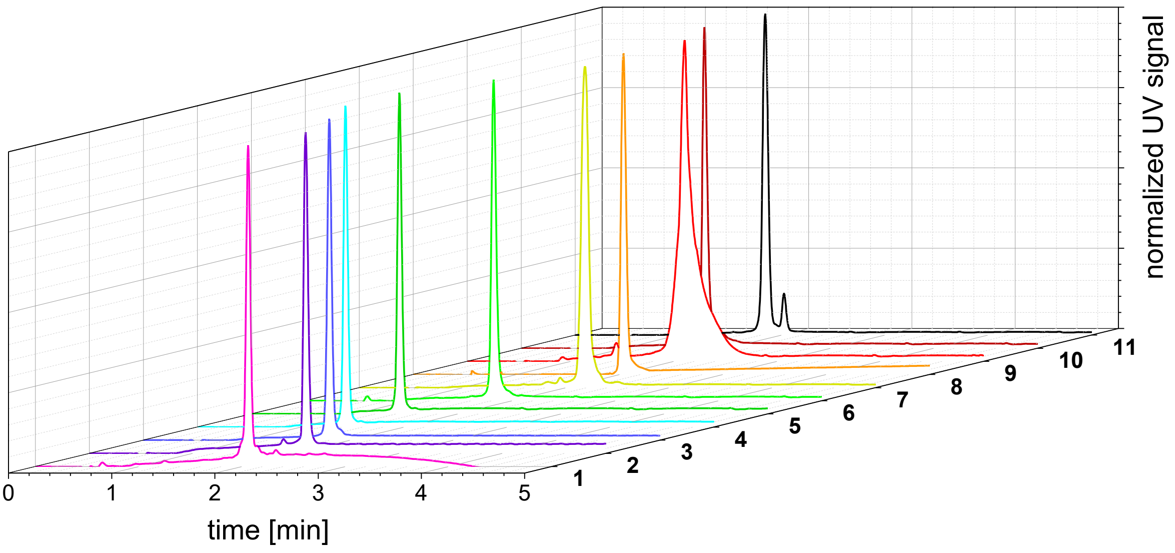

Figure S1: Analytical HPLC chromatograms of **1 – 11** after purification.

Results of the determination of the stability of [^{68}Ga]Ga-1 – [^{68}Ga]Ga-11 in human serum (analytical radio-HPLC chromatograms of the tracers incubated for 0, 15, 30, 45, 60, 75 and 90 minutes with human serum)

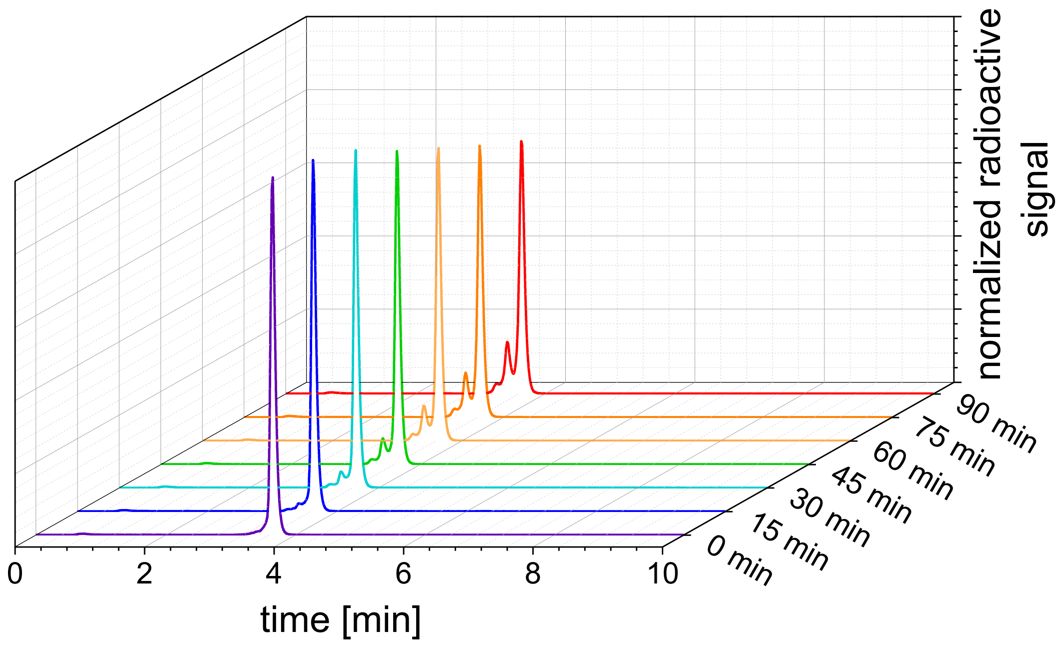

Figure S2: Analytical radio-HPLC chromatograms of [^{68}Ga]Ga-**1** at different time points of incubation with human serum.

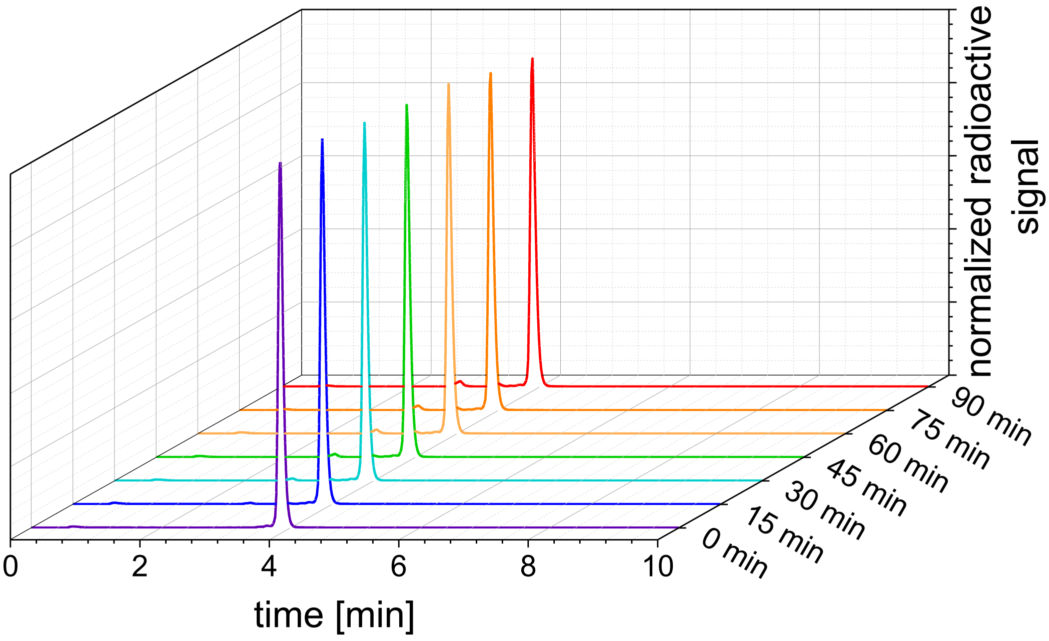

Figure S3: Analytical radio-HPLC chromatograms of [^{68}Ga]Ga-**2** at different time points of incubation with human serum.

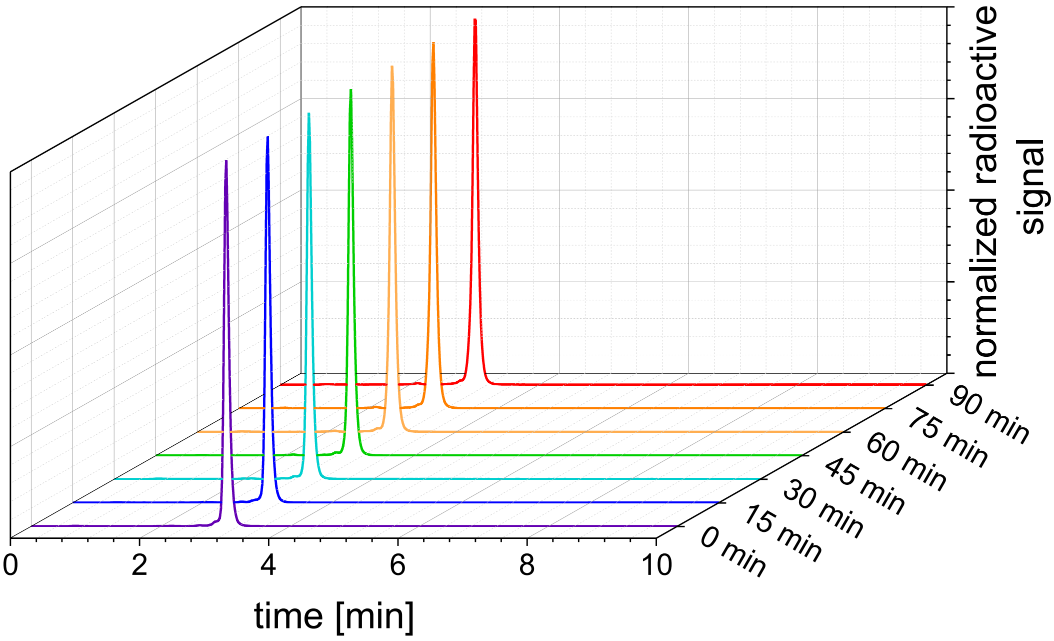

Figure S4: Analytical radio-HPLC chromatograms of [^{68}Ga]Ga-**3** at different time points of incubation with human serum.

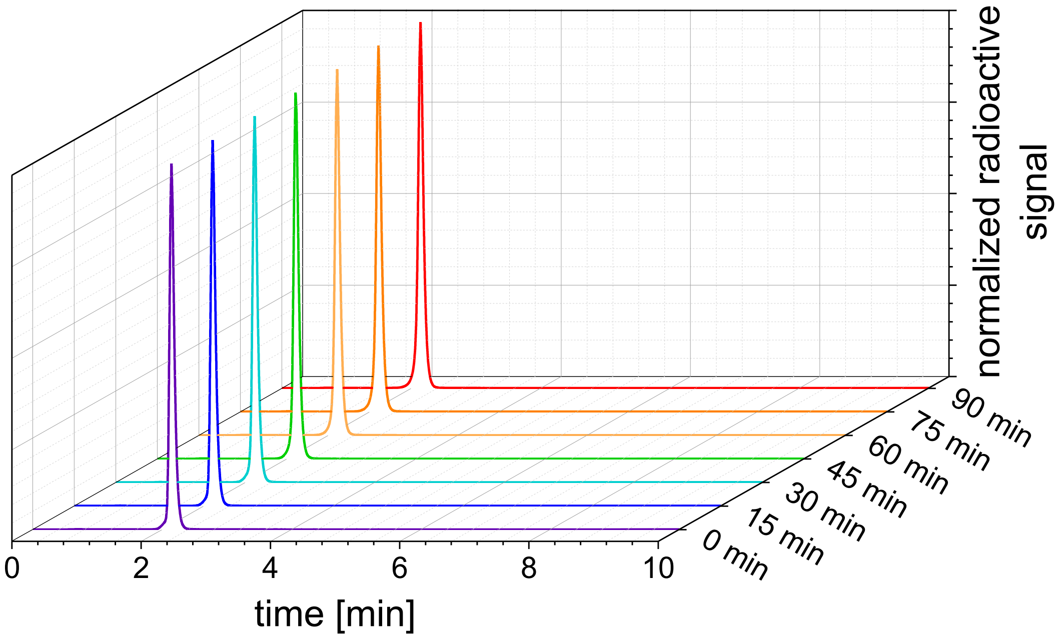

Figure S5: Analytical radio-HPLC chromatograms of [^{68}Ga]Ga-**4** at different time points of incubation with human serum.

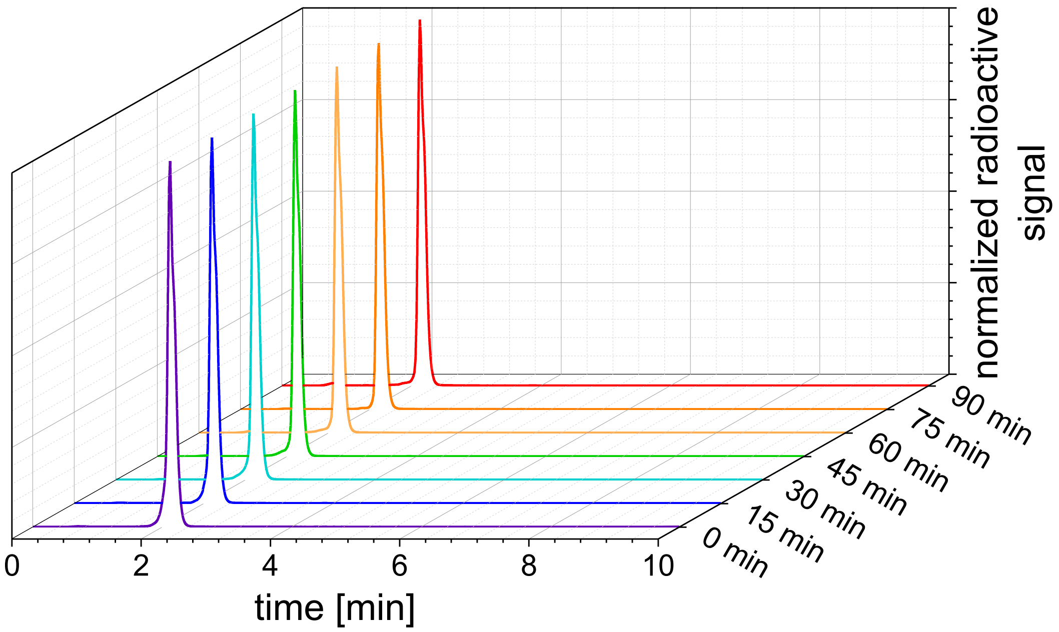

Figure S6: Analytical radio-HPLC chromatograms of [^{68}Ga]Ga-**5** at different time points of incubation with human serum.

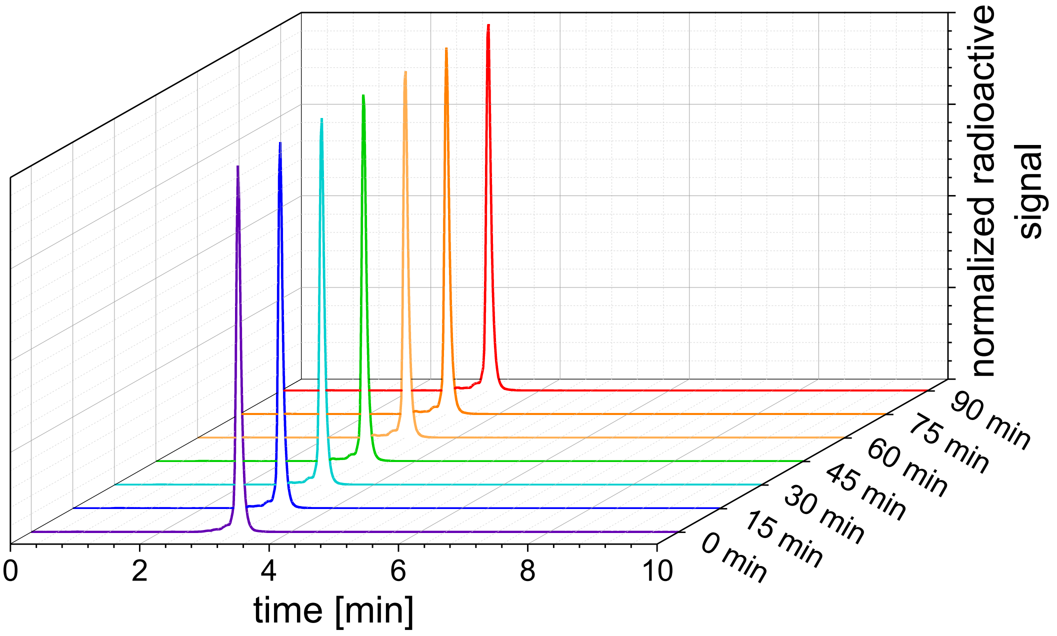

Figure S7: Analytical radio-HPLC chromatograms of [^{68}Ga]Ga-**6** at different time points of incubation with human serum.

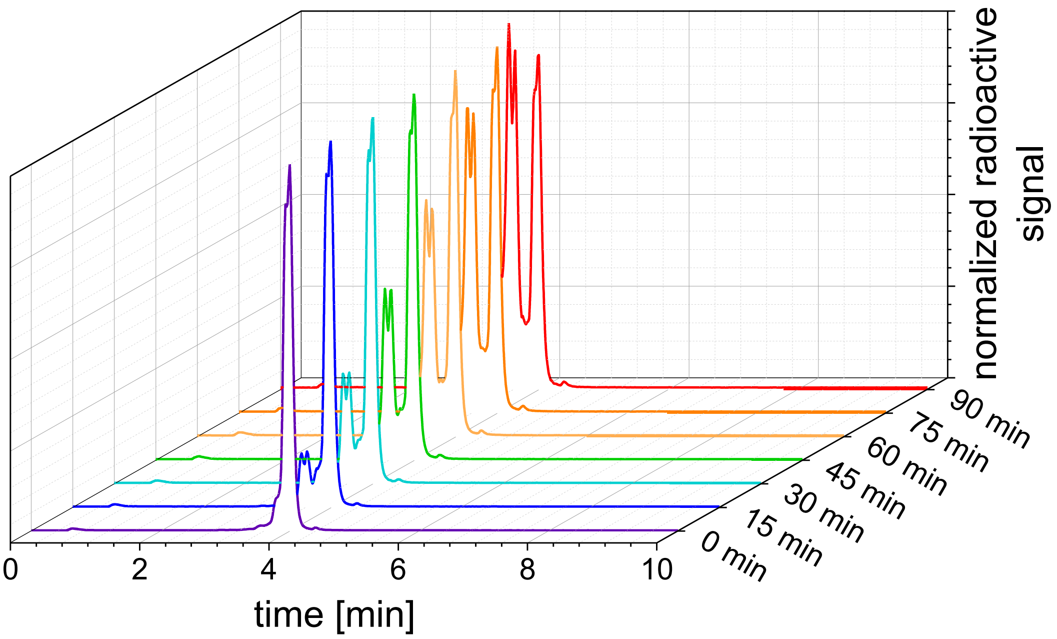

Figure S8: Analytical radio-HPLC chromatograms of [^{68}Ga]Ga-**7** at different time points of incubation with human serum.

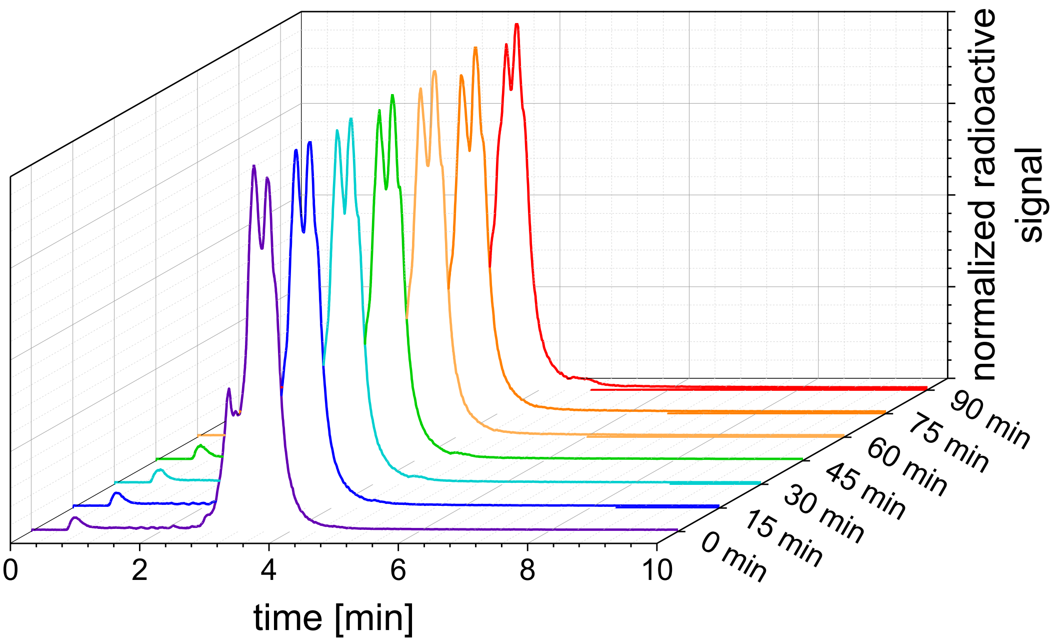

Figure S9: Analytical radio-HPLC chromatograms of [^{68}Ga]Ga-**8** at different time points of incubation with human serum.

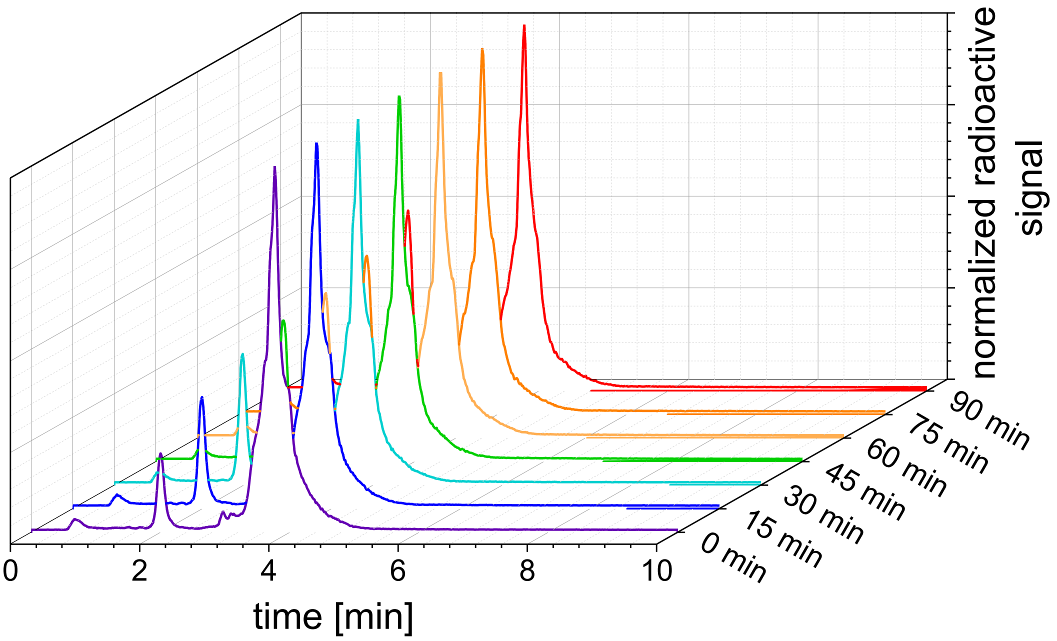

Figure S10: Analytical radio-HPLC chromatograms of [^{68}Ga]Ga-**9** at different time points of incubation with human serum.

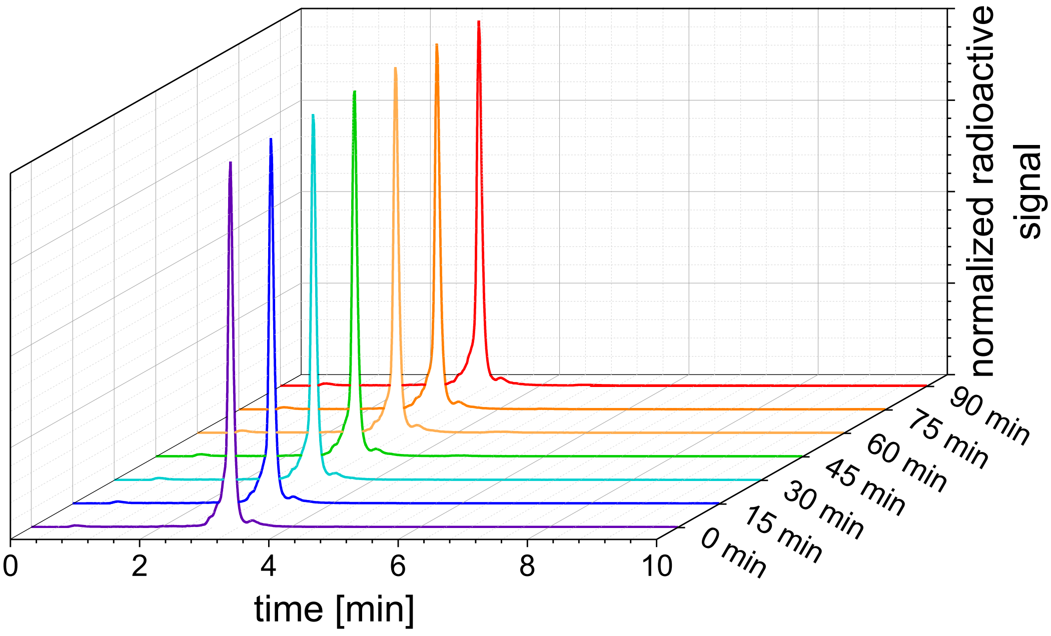

Figure S11: Analytical radio-HPLC chromatograms of [^{68}Ga]Ga-**10** at different time points of incubation with human serum.

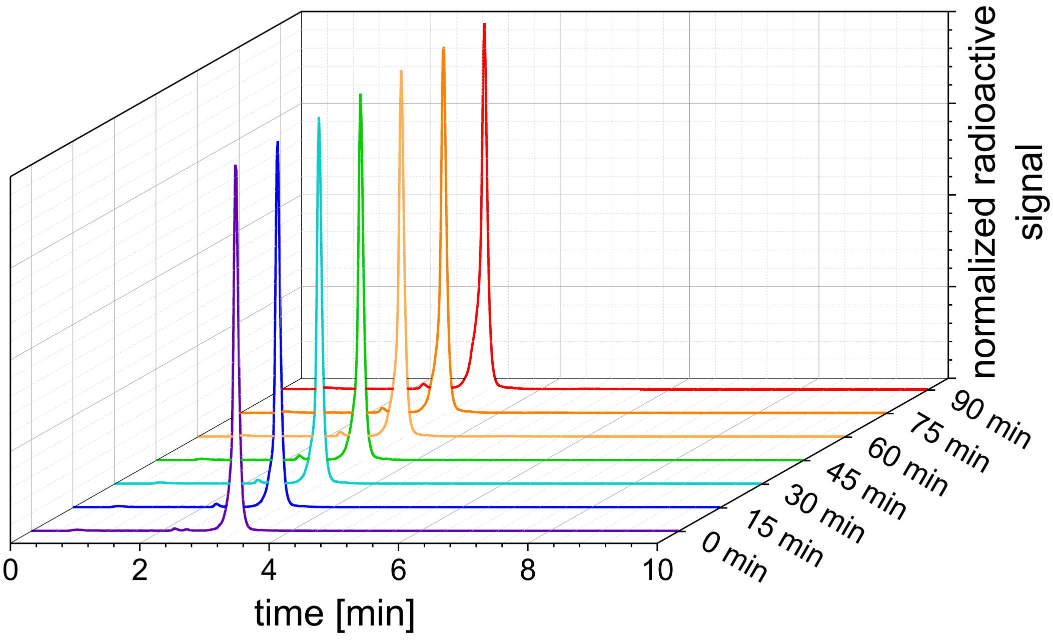

Figure S12: Analytical radio-HPLC chromatograms of [^{68}Ga]Ga-**11** at different time points of incubation with human serum.
